# A nanobody inhibitor of Fascin-1 actin-bundling activity and filopodia formation

**DOI:** 10.1098/rsob.230376

**Published:** 2024-03-20

**Authors:** Selena G. Burgess, Nikki R. Paul, Mark W. Richards, James R. Ault, Laurie Askenatzis, Sophie G. Claydon, Ryan Corbyn, Laura M. Machesky, Richard Bayliss

**Affiliations:** ^1^ Astbury Centre for Structural Molecular Biology, School of Molecular and Cellular Biology, Faculty of Biological Sciences, University of Leeds, Leeds LS2 9JT, UK; ^2^ Cancer Research UK Beatson Institute, Garscube Estate, Switchback Road, Bearsden, Glasgow G61 1BD, UK; ^3^ Institute of Cancer Sciences, University of Glasgow, Glasgow G61 1QH, UK; ^4^ Department of Biochemistry, University of Cambridge, Cambridge CB2 1GA, UK

**Keywords:** nanobody, Fascin-1, actin, cell migration, actin-bundling inhibitor

## Abstract

Fascin-1-mediated actin-bundling activity is central to the generation of plasma membrane protrusions required for cell migration. Dysregulated formation of cellular protrusions is observed in metastatic cancers, where they are required for increased invasiveness, and is often correlated with increased Fascin-1 abundance. Therefore, there is interest in generating therapeutic Fascin-1 inhibitors. We present the identification of Nb 3E11, a nanobody inhibitor of Fascin-1 actin-bundling activity and filopodia formation. The crystal structure of the Fascin-1/Nb 3E11 complex reveals the structural mechanism of inhibition. Nb 3E11 occludes an actin-binding site on the third β-trefoil domain of Fascin-1 that is currently not targeted by chemical inhibitors. Binding of Nb 3E11 to Fascin-1 induces a conformational change in the adjacent domains to stabilize Fascin-1 in an inhibitory state similar to that adopted in the presence of small-molecule inhibitors. Nb 3E11 could be used as a tool inhibitor molecule to aid in the development of Fascin-1 targeted therapeutics.

## Graphical abstract

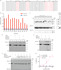

## 1. Introduction

Fascin-1 is an actin-binding protein that promotes the assembly of actin filaments into parallel bundles. Fascin-1-dependent bundling of F-actin underlies the formation of several classes of plasma membrane protrusions that drive cell migration, including finger-like filopodia and broad, flat lamellipodia that advance the leading edge of a migrating cell [1–4]. Fascin-1 is also involved in the formation and dynamics of podosomes and invadopodia which facilitate cellular invasion of the extracellular matrix [5–7]. Fascin-1 is a 55 kDa globular protein composed of four tandem β-trefoil domains that pack together to form a compact structure approximately 6 nm in diameter. It has at least two actin-binding surfaces and in filopodia, Fascin-1 cross-links parallel actin filaments into a hexagonal lattice, forming rigid bundles approximately 140 nm in diameter [8–12]. Phosphorylation of Fascin-1 at Ser^39^ by protein kinase C (PKC) negatively regulates its actin-binding activity and has been suggested to contribute to the highly dynamic nature of filopodial bundles [13–15]. Actin-based plasma membrane protrusions are crucial in embryonic development, for the function of motile cell types such as macrophages, and in processes such as wound healing [1–3]. However, in tumour cells, these structures enable metastasis and are frequently dysregulated in metastatic cancer, with greater size, longevity and number of protrusions associated with increased invasiveness. Fascin-1 expression, which is undetectable in normal adult epithelia [16], is upregulated in multiple cancer types and is often correlated with metastatic spread and poor patient outcome [17–20]. Fascin-1 is therefore of interest as a prognostic indicator and a therapeutic target. Small-molecule inhibitors of Fascin-1 are in development and have been used to target cancer cell invasion *in vitro* and in model systems [10,21–27]. The first Fascin-1 inhibitor to reach clinical trials was NP-G2-044. This compound appeared to exhibit some anti-tumour and anti-metastatic activity in a Phase 1 trial, although these improvements were not significant according to RECIST guidelines [28]. Such developments confirm that inhibition of Fascin-1 is a promising avenue and that more research is needed to find new ways to target Fascin-1 with greater efficacy.

Heavy chain only antibodies (HcAbs), which are produced by camelids and cartilaginous fish, lack light chains and bind antigens through a single variable domain on each heavy chain, known in camelids as VHH domains [29]. Within VHH domains, antigen binding primarily occurs through three variable complementarity determining regions (CDRs), which are separated by four constant framework regions (FRs). VHH domains can be isolated from HcAbs to derive single-domain antibodies (sdAbs). sdAbs are stable, monomeric protein reagents of approximately 15 kDa in size that can be produced in bacterial expression systems. Because *in vitro* screening methods can be used to isolate examples that have high affinity and specificity for an antigen of interest and any other properties that are required, camelid sdAbs, also known as nanobodies (Nbs), are increasingly being exploited in multiple applications, such as markers in high-resolution microscopy studies [30–32], in the development of biosensors [33], in medical imaging with potential for conjugation to radionuclides/photodynamic agents for targeted therapy [34,35], and as crystallization chaperones to stabilize conformationally flexible proteins [36–38]. Furthermore, there is considerable interest in the therapeutic potential of Nbs as inhibitors of the activities and interactions of target proteins, particularly because Nbs can interact with cryptic, shallow epitopes on antigens that are difficult to target by traditional small-molecule drug discovery methods [39–42]. Indeed, caplacizumab is the first Nb approved for clinical use to treat acquired thrombotic thrombotcytopenic purpura [43].

Here, we have sought Nb inhibitors of Fascin-1 and report the identification of Fascin-1-specific Nbs by phage display biopanning and the isolation of Nb 3E11 which acts as a potent inhibitor of Fascin-1 actin-bundling activity. A crystal structure of Nb 3E11 in complex with Fascin-1, combined with mass spectrometry methods, demonstrates the mechanism of Fascin-1 inhibition by Nb 3E11, which is distinct from that of current small-molecule inhibitors, providing new insight into how Fascin-1 inhibition can be achieved.

## 2. Results

### 2.1. Isolation of Fascin-1-specific nanobodies

Two llama nanobody phagemid libraries were generated using the peripheral blood mononuclear cell (PBMC) RNA from animals immunized with purified recombinant human Fascin-1 1-493. DNA sequencing of random clones from the libraries confirmed the presence of functional VHH coding sequences in greater than 80% of phagemid constructs. The total number of clones in each library was 1.17 × 10^9^ for library 1 and 1.22 × 10^9^ for library 2.

**Figure 1.**
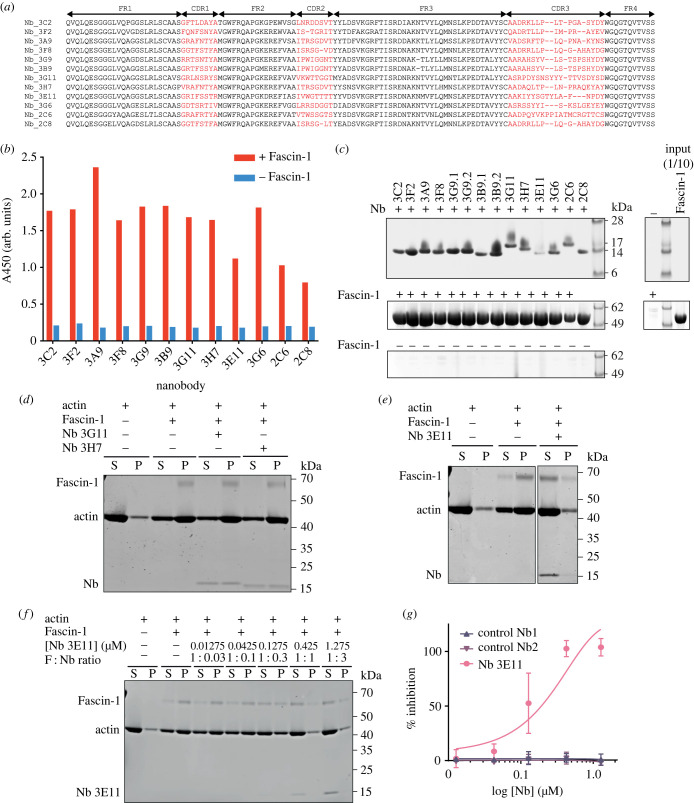
Identification and characterization of Fascin-1 specific Nbs. (*a*) Sequence alignment of Fascin-1-specific Nbs. CDR regions are coloured red. Nb sequence regions are classified according to the IMGT standard [44]. The N-terminal ‘MA’ residues and C-terminal His-tag present on each Nb are not shown. (*b*) Phage-nanobody ELISA results for Fascin-1 Nbs. Red bars represent reactions on wells coated with Fascin-1. Blue bars represent negative control reactions where no target was immobilized on wells. Representative of *N* = 2 independent experiments. (*c*) SDS-PAGE analysis of co-precipitation assay between Fascin-1 and immobilized Nbs. *N* = 2 independent experiments. (*d,e*) SDS-PAGE analysis of F-actin-bundling co-sedimentation assays with Fascin-1 and nanobodies 3G11, 3H7 and 3E11. *N* = 2 independent experiments. S, supernatant; P, pellet. (*f*) Representative SDS-PAGE gel from F-actin-bundling co-sedimentation assay with Fascin-1 and Nb 3E11. F, Fascin-1; S, supernatant; P, pellet. (*g*) Dose response curve of Fascin-1 F-actin-bundling activity in the presence of Nb 3E11, and control Nbs 1 and 2. Percentage inhibition of F-actin-bundling was quantified by densitometry of SDS-PAGE gels shown in (*f*) and electronic supplementary material, figure S2*b*,*c*. *N* = 3 biological replicates per condition. Error bars = standard deviation.

Three rounds of phage display biopanning were carried out with increasing stringency to isolate high affinity Fascin-1 nanobodies. Enrichment over the negative control (pan with no Fascin-1 immobilized) was observed (electronic supplementary material, table S1). Forty-four positive clones for each library pan output were randomly selected and used to express phage-nanobodies in *E. coli* TG1 and extracted by osmotic shock. The periplasm extracts were used as the input for streptavidin-based ELISAs using immobilized biotinylated Fascin-1. Fascin-1-specific nanobodies were identified as clones for which binding was observed to Fascin-1-coated wells and not the negative control reaction, where no Fascin-1 was immobilized (results for Pan Round 3 are shown in electronic supplementary material, figure S1*a*). One hundred and ninety-six positive clones from both libraries were sequenced and classified into 12 families based upon sequence identity in the CDRs (figure 1*a*). The phage-nanobody ELISA results for representatives of each sequence family are shown in figure 1*b*.

### 2.2. Biochemical characterization of Fascin-1-specific nanobodies

The selected representatives of each sequence family were recombinantly expressed as C-terminally His-tagged Nb proteins in the periplasm of *E. coli* SS320 and purified to homogeneity. Size-exclusion chromatography (SEC) revealed that Nbs 3B9 and 3G9 each displayed two populations (electronic supplementary material, figure S1*b*) corresponding to dimer and monomer protein. These two sub-populations were kept separate for further characterization.

*In vitro* co-precipitation assays with purified Nbs confirmed that all Nbs bound to Fascin-1 (figure 1*c*). Actin-bundling co-sedimentation assays revealed that 13 of the 14 Nbs did not negatively modulate the actin-bundling activity of Fascin-1 (figure 1*d* and electronic supplementary material, figure S1*c*–*h*). However, inclusion of Nb 3E11 resulted in a marked shift in Fascin-1 and actin distribution from the polymerized actin pellet fraction into the supernatant, indicating inhibition of Fascin-1-mediated actin-bundling by Nb 3E11 (figure 1*e*).

### 2.3. Nb 3E11 exhibits dose-dependent inhibition of Fascin-1-mediated actin-bundling

Titration of the Nb 3E11 : Fascin-1 molar concentration ratio in co-sedimentation assays demonstrated that Nb 3E11 acted as a dose-dependent inhibitor of Fascin-1 actin-bundling activity, achieving 100% inhibition at an equimolar concentration. In addition, Nb 3E11 at high concentrations shifted into the supernatant fraction together with actin and Fascin-1 (figure 1*f*,*g*).

To ensure that the CDR sequences of Nb 3E11 were responsible for the inhibitory action of the protein, control Nbs were constructed in which the CDRs were exchanged for unrelated CDRs (taken from Nbs specific to bacterial thermophilic proteins) while retaining the constant FRs of Nb 3E11. Two different control Nbs did not show observable binding to Fascin-1 in co-precipitation assays (electronic supplementary material, figure S2*a*), indicating the specific role of the Nb 3E11 CDR sequences. When these constructs were subsequently used as negative controls in actin-bundling assays and experiments in cells, they did not affect Fascin-1 actin-bundling activity (figure 1*g* and electronic supplementary material, figure S2*b*,*c*).

### 2.4. Structural basis of the Fascin-1/Nb 3E11 interaction

**Figure 2.**
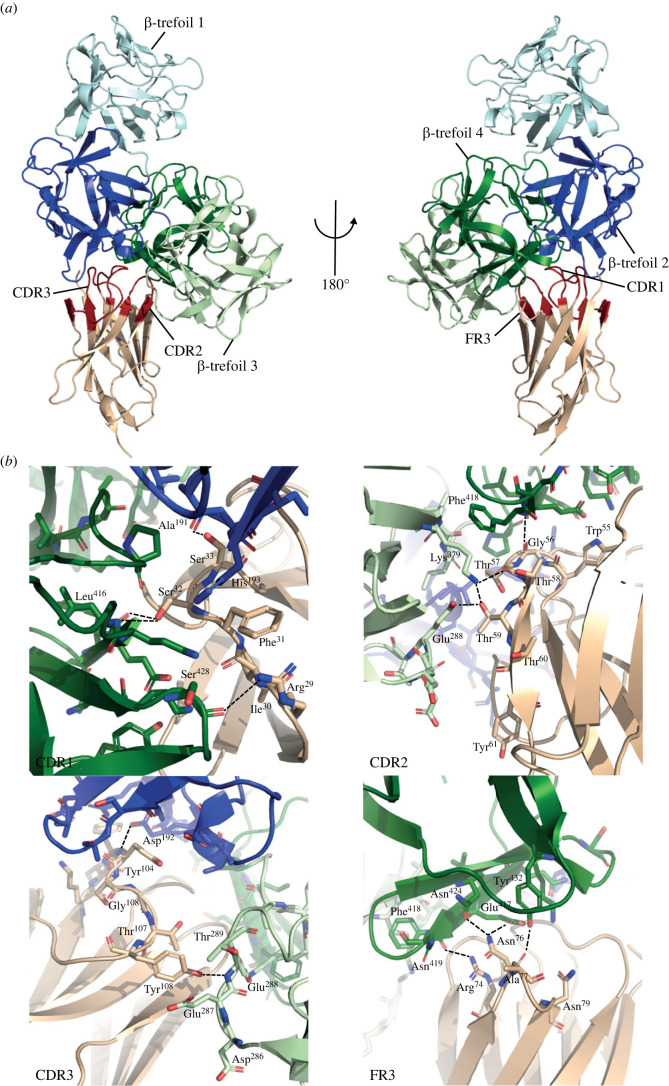
Crystal structure of the Fascin-1/Nb 3E11 complex. (*a*) Cartoon representation of the Fascin-1/Nb 3E11 structure. Fascin-1 is coloured blue and green with β-trefoil domains individually coloured: β-trefoil 1 (light blue), β-trefoil 2 (dark blue), β-trefoil 3 (light green), β-trefoil 4 (dark green). Nb 3E11 is coloured beige with CDRs shown in red. (*b*) Cartoon and stick representations of the binding interfaces between Fascin-1 (coloured as in *a*) and Nb 3E11 CDR1, CDR2, CDR3 and FR3 (shown in beige).

A structure of the Fascin-1 1-493/Nb 3E11 complex was resolved by X-ray crystallography at 2.2 Å resolution (table 1 and figure 2*a*). There are four copies of a 1 : 1 complex of Fascin-1/Nb 3E11 in the asymmetric unit (ASU) (electronic supplementary material, figure S3*a*). The other three complexes in the ASU display an overall C*α* RMSD of 0.19–0.27 Å when compared to the chain A/B complex (electronic supplementary material, figure S3*b*). Unambiguous electron density was observed for all chains (electronic supplementary material, figure S3*c*). Fascin-1 displays the four tandem β-trefoil folds observed in other structures [8,21]. Nb 3E11 adopts an immunoglobulin fold with a single disulfide bridge present and interacts directly with β-trefoils 2, 3 and 4 of Fascin-1 (figure 2*a*).

**Table 1.** Data collection and refinement statistics for Fascin-1/Nb 3E11.

	Fascin-1/Nb 3E11
*data collection*	
space group	*P*12_1_1
cell dimensions	
*a*, *b*, *c* (Å)	70.9, 210.3, 105.5
α, *β*, *γ* (°)	90.00, 91.26, 90.00
resolution range (Å)	105.16–2.20 (2.24–2.20)^a^
*R*_merge_ (%)	6.46 (61.1)
*I*/σ*I*	7.67 (1.14)
completeness (%)	99.86 (97.91)
redundancy	3.30 (2.80)
*refinement*	
resolution (Å)	59.44–2.20
no. reflections	149 244
*R*_work_/*R*_free_	24.68/29.04
no. atoms	
protein	18 301
hetero	99
water	944
mean *B*-factors	
protein	60.87
hetero	63.40
water	58.68
r.m.s. deviations	
bond lengths (Å)	0.003
bond angles (°)	0.671
*MolProbity analysis*	
all-atom clash-score	7.07
rotamer outliers (%)	0.11
Ramachandran outliers (%)	0
Ramachandran favoured (%)	93.88
MolProbity score	1.80

^a^Values in parentheses are for the highest resolution shell.

An average surface area of 841 Å^2^ per molecule is buried at the interface between Fascin-1 and Nb 3E11 (electronic supplementary material, figure S3*d*). All three CDRs and FR3 of Nb 3E11 form key interactions with Fascin-1 (figure 2*b*). From CDR1, the side-chains of Arg^29^ and Ser^32^ form hydrogen bonds with Fascin-1 β-trefoil 4 residues Ser^428^ and Leu^416^, respectively; an additional hydrogen bond is present between Ser^33^ of Nb 3E11 and Ala^191^ of β-trefoil 2; and the aromatic side-chain of Phe^31^ is stacked against His^193^. From CDR2, Thr^57^ and Thr^59^ form a hydrogen bond network involving Lys^379^ and Glu^288^ of β-trefoil 3 and the backbone of Gly^56^ forms a hydrogen bond with Phe^418^ of Fascin-1 β-trefoil 4. From CDR3, residues Tyr^104^ and Tyr^108^ hydrogen bond with Asp^192^ of β-trefoil 2 and Glu^288^ of β-trefoil 3, respectively. The constant region of Nb 3E11 also contributes directly to the interaction through residues Arg^74^ and Asn^76^ of FR3, which are central to a hydrogen bonding network involving Glu^417^, Phe^418^, Asn^424^ and Tyr^432^ of β-trefoil 4 (figure 2*b*).

**Figure 3.**
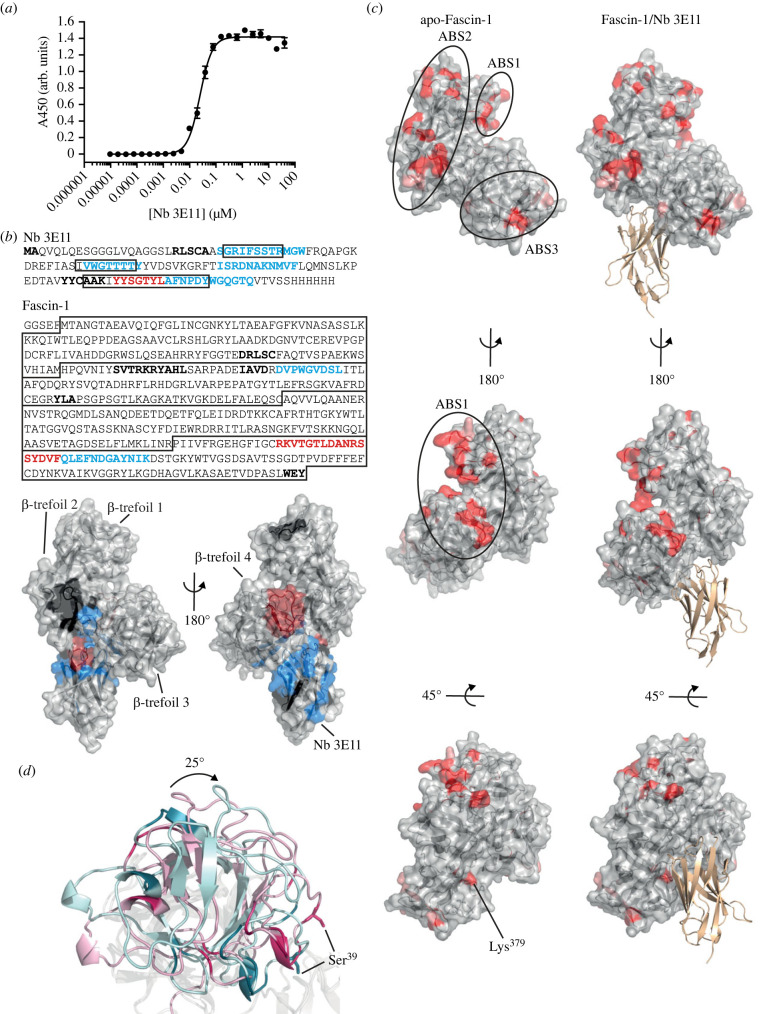
Mechanism of Fascin-1 inhibition by Nb 3E11. (*a*) ELISA binding assays between biotinyl-Fascin-1 (immobilized) and Nb 3E11. Responses were plotted against Nb 3E11 concentration and fitted to a one-site specific binding equation (solid line) in PRISM10 (GraphPad) to calculate binding affinity. *N* = 3 independent experiments. (*b*) HDX analysis of the Fascin-1/Nb 3E11 interaction. The amino acid sequences are shown for Nb 3E11 and Fascin-1; Nb 3E11 CDRs and Fascin-1 β-trefoil domains are marked with boxes. Residues that exhibit a substantial change in deuterium exchange upon complex formation are highlighted on the sequences of Fascin-1 and Nb 3E11, and on surface representations of the Fascin-1/3E11 crystal structure; increase in fractional uptake (red); decrease in fractional uptake (blue). Residues not covered in the HDX-MS analysis are shown in bold (black). Representative of *N* = 3 independent experiments. (*c*) Surface representations of apo-Fascin-1 (PDB 3P53) and Fascin-1/Nb 3E11 (Nb 3E11 is shown in cartoon representation). Mutation of residues coloured red has a substantial negative effect on Fascin-1 actin-bundling activity. Mutation of residues coloured pink has a moderate effect on Fascin-1 actin-bundling activity. Coloured residues are classified into ABS 1, 2 and 3 [9]. (*d*) Structural alignment of Fascin-1/Nb 3E11 and apo-Fascin-1 (PDB 3P53) with their β-trefoil 1 domains coloured light blue and light pink, respectively, and key actin-binding residues coloured dark blue and dark pink. The PKC phosphorylation site, Ser^39^, is indicated.

A binding affinity of 25 ± 0.9 nM between Nb 3E11 and Fascin-1 1-493 was measured by enzyme-linked immunosorbent assay (ELISA) (figure 3*a*). The strong binary interaction allowed hydrogen–deuterium exchange mass spectrometry (HDX-MS) to be used to validate the complex crystal structure by probing the changes in protein solvation associated with complex formation in solution. The sequence coverage of Fascin-1 was 95% and 90.2% for Nb 3E11 (figure 3*b*). Reduction in deuterium uptake upon complex formation represents increased protection and/or reduced solution dynamics of the residues involved and indicates association with protein–protein binding interfaces. Fascin-1 β-trefoil 2 peptide ^168^DVPWGVDSL^176^ and β-trefoil 4 peptide ^415^QLEFNDGAYNIK^426^, which overlap with the binding interface identified in the complex crystal structure, displayed reduced deuterium exchange in the presence of Nb 3E11. Similarly, Nb 3E11 peptides corresponding to CDR1 ^27^SGRIFSSTRMGW^38^, CDR2 ^54^VWGTTTTY^61^, CDR3 ^110^AFNPDYWGQGTQ^121^ and FR3 ^72^ISRDNAKNMVF^82^ all displayed a reduction in deuterium exchange upon interaction with Fascin-1, which correlated with the interface observed in the crystal structure (figure 2*b* and electronic supplementary material, figure S3*d*).

Residues displaying a substantial increase in deuterium uptake upon complex formation, implying increased solvent exposure, likely resulted from conformational changes induced in these regions concomitant with binding. Positive shifts in deuterium exchange rate upon complex formation were observed for Fascin-1 β-trefoil 4 peptide ^398^RKVTGTLDANRSSYDVF^414^, which is adjacent to the binding site identified by HDX-MS analysis and in the crystal structure, and Nb 3E11 CDR3 peptide ^103^YYSGTYL^109^, which is involved in direct binding to Fascin-1 but is only partially buried in the crystal structure (figure 2*b* and electronic supplementary material, figure S3*d*).

The strong correlation between the binding interfaces identified by HDX-MS analysis and those in the Fascin-1/Nb 3E11 crystal structure serve to validate the structural basis of the interaction observed by X-ray crystallography, providing a foundation on which to understand how Nb 3E11 inhibits Fascin-1 activity.

### 2.5. Mechanism of Fascin-1 inhibition by Nb 3E11

In previous work, mutagenesis of surface residues has been used to identify two conserved actin-binding sites (ABS) on Fascin-1. ABS1 and ABS2 are surface patches formed by coordination of β-trefoil 1 with β-trefoil 4 and β-trefoil 2, respectively. Mutations on β-trefoil 3, including K379A, also disrupt actin-bundling activity and have been suggested to fall within either a third ABS or an extension of ABS1 or ABS2 (figure 3*c*) [9].

Nb 3E11 acts to inhibit Fascin-1-mediated actin-bundling activity at multiple ABS. Fascin-1 proteins mutated within ABS1 or ABS3, and that exhibit a greater than 80% reduction in actin-bundling activity compared to WT Fascin-1, were further inhibited by addition of Nb 3E11 [9] (electronic supplementary material, figure S4*a*). The inhibitory activity of Nb 3E11 at ABS3 likely results from direct occlusion of Lys^379^ on β-trefoil 3 (figures 2*b* and 3*c*). The Fascin-1 K379A mutant retained the ability to bind Nb 3E11 (electronic supplementary material, figure S4*b*), which is unsurprising given the extensive buried surface area at the interface (electronic supplementary material, figure S3*d*).

Nb 3E11 also allosterically inhibits actin binding, by stabilization of a Fascin-1 conformation in which ABS1 and ABS2 are both distorted (figure 3*c*). HDX-MS showed that the β-trefoil 4 peptide ^398^RKVTGTLDANRSSYDVF^414^, which contacts β-trefoil 1 to form the surface corresponding to ABS1, undergoes a substantial conformational change induced by binding of Nb 3E11 to an adjacent region of Fascin-1 (figure 3*b*). A comparison of the Fascin-1/Nb 3E11 complex crystal structure to the structure of Fascin-1 alone shows that this disruption of the intramolecular interaction between β-trefoil 4 and β-trefoil 1 in the presence of Nb 3E11 is accompanied by a 25° rotation of β-trefoil 1 so that all the actin-binding residues on β-trefoil 1 have altered positions relative to the remaining portions of ABS1 and ABS2 on the adjacent domains (figures 3*d* and 5*a*; electronic supplementary material, movie S1). Thus, the conformational change associated with Nb 3E11 binding to β-trefoil 4 distorts both of the principal actin-binding surfaces of Fascin-1. This allosteric effect, in conjunction with Nb binding directly occluding Lys^379^ on β-trefoil 3, explains the inhibitory effect of Nb 3E11 on Fascin-1 actin-bundling activity.

### 2.6. Nb 3E11 disrupts Fascin-1-mediated filopodia

**Figure 4.**
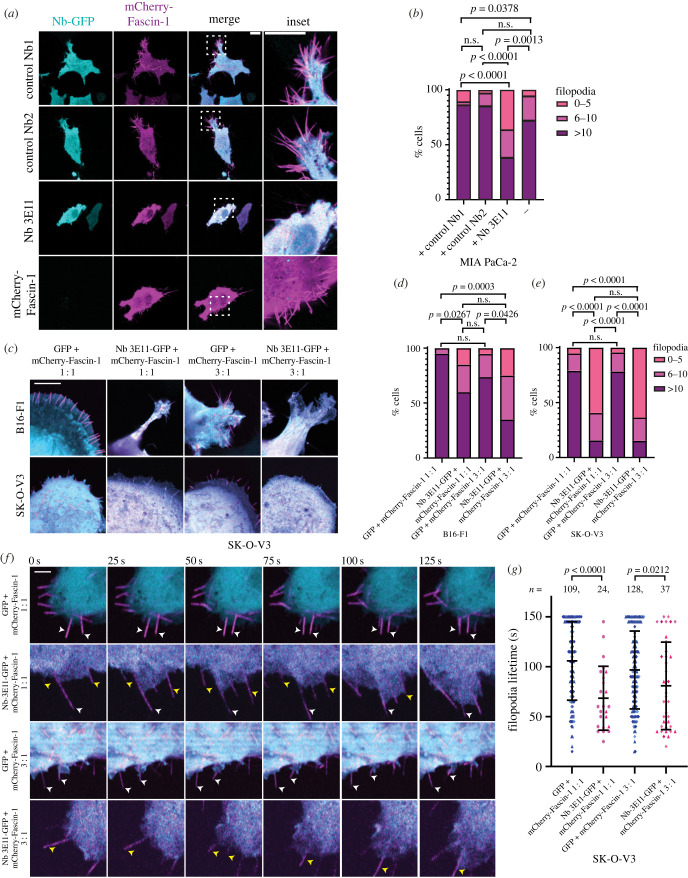
Nb 3E11 demonstrates dose-dependent inhibition of Fascin-1-mediated filopodia. (*a*) MIA PaCa-2 human pancreatic cancer cells expressing mCherry-Fascin-1 either alone or in combination with control Nb1-GFP, control Nb2-GFP or Nb 3E11-GFP, imaged by super-resolution confocal microscopy. Scale bars = 10 µm. White dashed boxes indicate inset areas. (*b*) Quantification of filopodia number in MIA PaCa-2 cells expressing mCherry-Fascin-1 alone, or in combination with control Nb1-GFP, control Nb2-GFP or Nb 3E11-GFP. *n* ≥ 34 cells per condition over 3 biological replicates prepared independently on different days. *P*-values as shown; *χ*^2^-test; n.s., not significant. (*c*) Super-resolution confocal microscopy of mCherry-Fascin-1 in B16-F1 and SK-OV-3 cancer cells expressing 1 : 1 or 3 : 1 ratios of GFP or Nb 3E11-GFP DNA constructs : mCherry-Fascin-1. Cyan, GFP or Nb 3E11-GFP; magenta, mCherry-Fascin-1. Scale bars = 10 µm. (*d*) Quantification of filopodia number in B16-F1 cells and (*e*) SK-OV-3 expressing 1 : 1 or 3 : 1 ratios of GFP or Nb 3E11-GFP DNA constructs : mCherry-Fascin-1. *n* = 19–33 cells per condition over at least 3 biological replicates prepared independently on different days. *P*-values as shown; *χ*^2^-test; n.s., not significant. (*f*) Live-cell super-resolution confocal microscopy of mCherry-Fascin-1 in SK-OV-3 cancer cells expressing 1 : 1 or 3 : 1 ratios of GFP or Nb 3E11-GFP DNA constructs : mCherry-Fascin-1. Cyan, GFP or Nb 3E11-GFP; magenta, mCherry-Fascin-1. Scale bars = 2 µm. Time in seconds as indicated. White arrows indicate stable filopodia, yellow arrows indicate dynamic filopodia. (*g*) Quantification of filopodia lifetime from movies shown in (*f*), imaged for 150 s. Filopodia tracked from movies generated over at least three biological replicates prepared independently on different days. *P*-values from complete dataset and *n* numbers of filopodia as shown; Mann–Whitney two-tailed *t*-test; n.s. = not significant. *P*-values were validated for unequal sample sizes by randomly down-sampling the GFP controls to match the *n* number of the Nb 3E11-GFP condition. Error bars = standard deviation. Different symbol colours represent values from different biological replicates.

To understand the effect of Nb 3E11 on filopodia, MIA PaCa-2 pancreatic cancer cells were transiently transfected with mCherry-tagged Fascin-1 and GFP-tagged Nbs. Images of live cells were acquired using confocal microscopy to avoid fixation artefacts. Cells were also transfected with Nbs alone to check for signal bleed-through between channels (electronic supplementary material, figure S5*a*). Quantification of the number of filopodia showed that expression of Nb 3E11-GFP led to a significant reduction in the number of Fascin-1-mediated filopodia generated by cells (figure 4*a*,*b*). Furthermore, these cells frequently appeared blebby (figure 4*a*,*c*). Expressing a higher ratio of Nb 3E11-GFP to mCherry-Fascin-1 in multiple cell lines led to increased, but not significant inhibition of filopodia formation, demonstrating that Nb 3E11 inhibits Fascin-1-dependent filopodia production *in cellulo* in a dose-dependent manner (figure 4*c*–*e* and electronic supplementary material, figure S5*b*,*c*). Nb 3E11 had no effect on cell proliferation over the duration of these experiments (electronic supplementary material, figure S6).

In addition to the paucity of filopodia in SK-OV-3 cells expressing Nb 3E11-GFP, the filopodia that remained exhibited significantly shorter lifetimes (figure 4*f*,*g* and electronic supplementary material, movie S2). This was not consistently observed in B16-F1 and MIA PaCa-2 cells (electronic supplementary material, figure S5*d*,*e*). These data indicate that Nb 3E11 expression reduces Fascin-1-mediated filopodia formation and stability in cells.

## 3. Discussion

### 3.1. Inhibition of Fascin-1 actin-bundling activity

**Figure 5.**
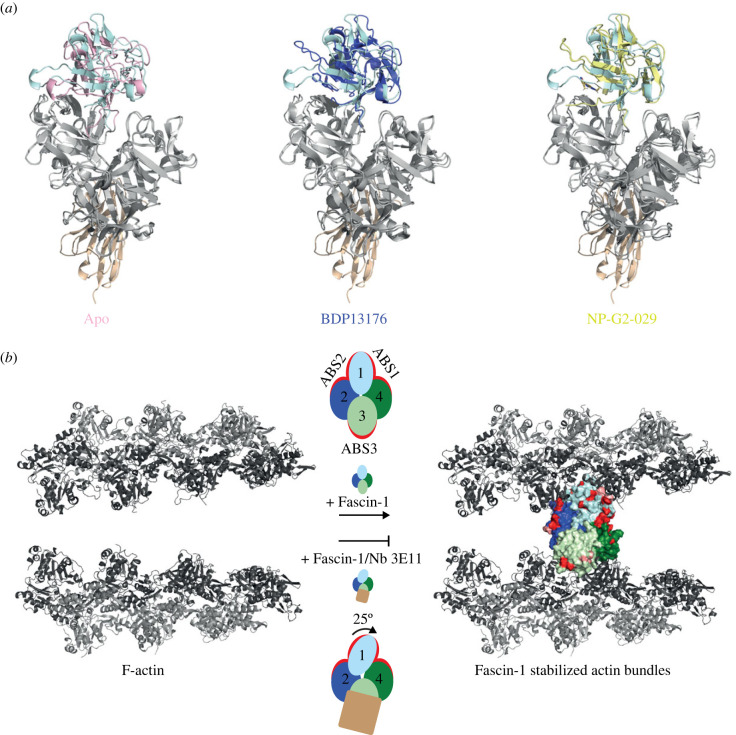
Inhibition mechanisms of Fascin-1 actin-bundling activity. (*a*) Structural alignments of Fascin-1 structures in cartoon representation. In each representation, Fascin-1 β-trefoils 2–4 are shown in grey, Nb 3E11 in beige, and Fascin-1 β-trefoil 1 domains are coloured as follows: Fascin-1/Nb 3E11 (light blue) aligned with apo-Fascin-1 (PDB 3P53) (pink); Fascin-1/Nb 3E11 (light blue) aligned with Fascin-1/BDP13176 (PDB 6I18) (purple); Fascin/Nb 3E11 (light blue) aligned with Fascin-1/NP-G2-029 (PDB 6B0T) (yellow). (*b*) Illustration of Fascin-1-mediated actin-bundling and its inhibition by Nb 3E11. F-actin (grey) is bundled by Fascin-1 forming bridges between adjacent filaments. Fascin-1 β-trefoils 1 (light blue) and 2 (dark blue) produce ABS2 (red), and β-trefoils 1 (light blue) and 4 (dark green) generate ABS1 (red), resulting in a double actin-binding region on one side of the molecule. Fascin-1 β-trefoil 3 (light green) forms a single actin-binding region, ABS3 (red), on the opposite side of the molecule. On binding of Nb 3E11 (beige), ABS3 is blocked directly, and ABS1 and 2 are distorted by a 25° rotation in Fascin-1 β-trefoil 1. Thus, Fascin-1/Nb 3E11 complexes are unable to bind to actin filaments on either side, and the bundling activity of Fascin-1 is abolished. Molecular coordinates for the Fascin-1/F-actin complex were kindly provided by X. Wu and G. Dong (Shantou University Medical College, China) [48].

Fascin-1 is a potent driver of metastasis and its overexpression is associated with the most aggressive human carcinomas [17,45]. Depletion of Fascin-1 or inhibition of its actin-bundling activity in cancer cells reduces tumour invasion and metastasis [25,46,47]. To date, efforts to inhibit Fascin-1 activity have focused on the development of small-molecule inhibitors targeting the cleft between β-trefoils 1 and 2 [10,22–24]. The inhibitors BDP-13176 and NP-G2-029 bind in this cleft, blocking ABS2 and inducing a rotation of β-trefoil 1 that also distorts ABS1 [22,24]. The compact arrangement of β-trefoil domains that is observed in crystal structures in the absence of inhibitors is considered to correspond to the actin-bound conformation in which the principal actin-binding surfaces are assembled at the interfaces between β-trefoil 1 and adjacent domains. Interfering with the position of β-trefoil 1 within this arrangement correlates with disruption of actin-bundling activity [22,24]. In the present work, we report the discovery of Nb 3E11, which distorted ABS1 and ABS2 through interaction with β-trefoil 4, a previously untargeted site for the inhibition of Fascin-1 actin-bundling activity, and directly occluded Lys^379^ on β-trefoil 3, which has been identified as an important ABS residue [9]. Nb 3E11 induced a conformational change in the region of β-trefoil 4 adjacent to β-trefoil 1 that caused β-trefoil 1 to be rotated into an inactive position, similar to the orientation adopted in the presence of small-molecule inhibitors (figure 5*a*). Nb 3E11 is therefore an example of a β-trefoil 3–4-directed inhibitor of Fascin-1 actin-bundling activity, providing evidence for the potential for new inhibitors to target other sites on Fascin-1 (figure 5*b*).

Although the conformations observed in crystal structures of inhibited or mutant Fascin-1 are substantially different from a conformation competent to bind actin, the protein in solution may also sample more open conformations through interdomain motions. FRET analysis of the conformational dynamics of Fascin-1 and fluorescence recovery after photobleaching (FRAP) analysis of the dynamics of Fascin-1 association with actin [49] suggest that Fascin-1 oscillates between a compact, actin-binding conformation and an open state upon phosphorylation at Ser^39^ by PKC (figure 3*d*). Molecular dynamics simulations of Fascin-1 mutants with impaired actin binding display different conformations and increased protein flexibility compared to WT Fascin-1 [50,51]. Furthermore, Fascin-1-dependent actin-bundling in functional filopodia is not characterized by stable cross-linking of actin fibres, but rather is a highly dynamic process involving rapid cycles of actin binding and disassociation. Because conformational and actin-binding dynamics appear to be important for the activity of Fascin-1, the restriction of interdomain movements imposed by binding of Nb 3E11 at an epitope incorporating parts of β-trefoils 2, 3 and 4 may also contribute to its inhibitory activity.

Nb 3E11 was observed to inhibit Fascin-1-dependent filopodia formation in several cell lines in a dose-dependent manner, indicating that the Nb 3E11 : Fascin-1 ratio is pertinent to observation of *in cellulo* inhibition of Fascin-1 function. The epithelial ovarian adenocarcinoma cell line SK-OV-3 was more sensitive to Nb 3E11-mediated inhibition of Fascin-1 than the neural crest derived B16-F1 (mouse melanoma) and MIA PaCa-2 (pancreatic carcinoma) cells lines, which both exhibit a mixed and dynamic morphology in culture, suggesting that the cytoskeletal phenotype of the cells may determine the efficacy of Fascin-1 inhibition [52].

### 3.2. Evidence for ABS3 on Fascin-1

An outstanding question regarding the actin-bundling activity of Fascin-1 is whether it possesses a third ABS. Alanine-scanning mutagenesis of Fascin-1 surface residues has identified a patch of residues that are important for actin-bundling but which lay on β-trefoil 3, a considerable distance from either ABS1 or ABS2 [9]. Based on cryo-electron tomography data, a model of the Fascin-1 complex with F-actin has been proposed in which Fascin-1 ABS 1 and 2 form a double actin-binding site on one side of the protein and ABS3 provides a single actin-binding site on the opposing side, enabling bundling of two adjacent actin filaments [48,53]. Nb 3E11 occludes Lys^379^, which is a key residue within this putative third ABS, and we suggest that this direct mechanism may contribute to the inhibitory effect of Nb 3E11 on Fascin-1 actin-bundling activity. Further work is required to clarify the role of ABS3 and whether it can be exploited in the development of Fascin-1 inhibitors.

### 3.3. Applications of single-domain antibodies

Single-domain antibody (sdAb) technology has the potential to provide targeted inhibitors by an alternative pathway to traditional small-molecule drug discovery. sdAbs are small, soluble, extremely stable antibody domains, they bind to cryptic shallow epitopes that are rarely sampled with high affinity by traditional methods, and they can be isolated in a relatively short time. Indeed, sdAbs can be used directly as biological therapeutics for extracellular targets as exemplified by the success of caplacizumab for the treatment of acquired thrombotic thrombotcytopenic purpura [43]. However, like most proteins, sdAbs are too large and hydrophilic to pass readily across cell membranes. Many strategies have been explored to overcome this and facilitate the delivery of sdAbs into the interior of cells in a manner that might work outside the laboratory context: adjustment of surface charge through substitution of residues in the invariant regions for basic amino acids or addition of cell-penetrating tags or other carriers have been shown to enhance cellular uptake [54,55], the robustness of sdAb proteins may make them suitable for intracellular delivery as cargo by lipid nanoparticles [56], and introduction of sdAbs at the nucleic acid level is an emerging therapeutic possibility [57]. However, reliable means for their delivery across cell membranes still have to be developed before sdAbs directed against intracellular targets, such as Nb 3E11, can be used directly as therapeutics. Nevertheless, such sdAbs may identify sites of protein–protein interaction or new potential sites of target inhibition by acting as competitor molecules either directly or allosterically *in vitro*. This potential is demonstrated by Nb 3E11, which efficiently inhibited Fascin-1 actin-bundling activity by binding at a previously untargeted site on Fascin-1. In addition to Nb 3E11, we identified several Nbs which bound to Fascin-1 without altering its actin-bundling activity. Similarly, a previously identified Nb called FASNb5 alters Fascin-1 actin bundle morphology without affecting bundling activity [58,59]. Such reagents may be useful for staining in super-resolution imaging methods which benefit from the smaller size of sdAbs compared to IgGs [30,60,61].

In conclusion, we have isolated and characterized Nb 3E11, a potent sdAb inhibitor of Fascin-1 actin-bundling activity and filopodia formation. Nb 3E11 has potential for use as a tool for Fascin-1 inhibition in cells and to guide development of small-molecule or peptide inhibitors targeting a previously unexploited site on the protein.

## 4. Material and methods

### 4.1. Protein expression and purification

Full-length human Fascin-1 WT and mutants were expressed in CodonPlus RIL *E. coli* BL21(DE3) cells and purified as described previously [22]. The protein was stored in 10 mM Tris pH 7.5, 50 mM NaCl. A version engineered to incorporate an N-terminal Avi-tag sequence was co-expressed with pBirAcm (Avidity LLC) in *E. coli* B834 cells using media supplemented with biotin in order to generate selectively biotinylated material, which was then purified in a similar manner. C-terminally His-tagged Nbs were expressed in the periplasm of *E. coli* SS320 cells transformed by pBLIP1 and purified by nickel affinity chromatography and SEC on a Superdex 75 X16/600 column as previously described [62].

### 4.2. Library generation and phage display biopanning

Two llamas were inoculated with 300 µg Fascin-1 1-493 at four intervals over a two-month period. RNA was extracted from the PBMC fraction isolated from blood samples taken from each animal. These procedures were carried out by Preclinics GmbH. cDNA synthesis was performed using the IgG-specific primer CALL002 (5′ ggtacgtgctgttgaactgttcc 3′). Library construction was performed as described previously [62] with the VHH coding regions ligated into the phagemid vector pBLIP1 and used to transform *E. coli* TG1 (Lucigen) by electroporation.

Library rescue and phage display biopanning were performed as described previously [62,63] using biotinyl-Avi-tagged Fascin 1-493 as the immobilized target. To identify clones specific to Fascin-1, small-scale phage-nanobody expressions were carried out and binding was assessed by ELISA [62]. Phagemids encoding Nbs that specifically bound Fascin-1 were purified and sent for DNA sequencing (Eurofins Genomics).

### 4.3. Molecular biology

K41A, K358A and K379A mutations were introduced into the WT Fascin-1 coding sequence using the QuikChange method and the following primers:

K41A_F, 5′ gaacgcgtccgccagcagcctggcgaagaagcagatctggacgctgg 3′,
K41A_R, 5′ ccagcgtccagatctgcttcttcgccaggctgctggcggacgcgttc 3′;
K358A_F, 5′ caatggcaagtttgtgacctccgcgaagaatgggcagctggccgcctc 3′,
K358A_R, 5′ gaggcggccagctgcccattcttcgcggaggtcacaaacttgccattg 3′;
K379A_F, 5′ ggactcagagctcttcctcatggcgctcatcaaccgccccatcatcg 3′,
K379A_R, 5′ cgatgatggggcggttgatgagcgccatgaggaagagctctgagtcc 3′.

The R149A, K150A, R151A triple mutation was introduced using the inverse PCR mutagenesis method with the primers RAKARA_F, 5′ cggcctacgcgcacctgagcgcgcggccggccgacgagatcgccgtg 3′ and RAKARA_R, 5′ cagcggtgacgctgtagatgttgacctgagggtgcatggcgatgtgc 3′. Fascin-binding Nb sequences were amplified from pBLIP1 phagemids by PCR and inserted between the XhoI and BamHI sites of pEGFP (Clontech) to generate C-terminally GFP-tagged Nb constructs for mammalian expression. Equivalent control constructs were made by replacing the CDRs of Nb 3E11 with CDR sequences from previously identified Nbs against unrelated bacterial proteins. The CDRs of Control Nb1 were taken from a Nb selective for a *Deinococcus geothermalis* fumarate transporter [64]. The CDRs of Control Nb2 were taken from an Nb selective for SbsB protein from *Geobacillus stearothermophilis* [65].

### 4.4. Co-precipitation assays

*In vitro* recombinant protein co-precipitation assays were performed as described previously using Nbs immobilized on Nickel Sepharose as the bait proteins for untagged Fascin-1 [63].

### 4.5. F-actin-bundling assays

F-actin was generated by polymerization of 50 µM rabbit skeletal muscle G-Actin (greater than 95% purity, Cytoskeleton, Inc.) in 5 mM Tris pH 8.0, 0.2 mM CaCl_2_, 0.2 mM ATP (G-Buffer) supplemented with 100 mM KCl for 1 h at 26°C. Fascin-1 protein was diluted to 0.5 µM in G-Buffer + 100 mM KCl and incubated with equimolar concentrations of Nb proteins for 30 min at 26°C, unless otherwise stated. F-actin was then added to a final concentration of 5 µM and the reaction was incubated for a further 30 mins at 26°C. The mixture was centrifuged at 8000*g* for 20 min at room temperature (RT). The supernatant and pellet were separated, and the pellets were re-suspended in 100 µl of G-Buffer. Supernatant and pellet fractions were mixed with reducing SDS sample buffer and separated by SDS-PAGE on 4–12% Bis-Tris gels. Gels were stained with InstantBlue Protein Stain (Expedeon) and scanned using a Li-Cor Odyssey CLx Scanner. The images were analysed using ImageStudio software (v. 5.2).

### 4.6. X-ray crystallography

To produce the complex for crystallization trials, Fascin-1 and Nb 3E11 were mixed at 1 : 1.2 stoichiometry and subjected to SEC on a Superdex 200 X16/600 column (Cytiva) equilibrated in 10 mM Tris pH 7.5, 50 mM NaCl. Fractions containing the complex were concentrated to 20 mg ml^−1^ and mixed 1 : 1 with well solution in crystallization trials performed by vapour diffusion at 295 K. A single crystal was obtained with Morpheus screen condition F2 (Molecular Dimensions) (0.02 M d-glucose, d-mannose, d-galactose, l-fucose, d-xylose, *N*-acetyl-d-glucosamine; 0.1 M MES/imidazole pH 6.5, 10% PEG 8000, 20% ethylene glycol) and frozen directly in liquid nitrogen.

Data were collected on beamline IO4 at Diamond Light Source (Oxford, UK) and processed by the DIALS automatic software pipeline. phenix.xtriage [66] identified the presence of pseudotranslation between molecules related by non-crystallographic symmetry in the data. Phasing was performed by Molrep [67] using trefoils 2–4 of Fascin-1 (PDB 3LLP [21]) and a Gelsolin nanobody (PDB 2X1O [68]) as models. A pseudotranslation vector (0.000, 0.013, 0.500) was applied during molecular replacement. Iterative rounds of manual model building and refinement were carried out using phenix.refine [66,69] and Coot [70]. Zanuda was used to check space group assignment [71]. Structure validation was performed by Molprobity [72].

### 4.7. ELISA assays

ELISA assays were performed as described previously [73]. The HRP-conjugated anti-His-tag antibody (ab1187; Abcam) was used to resolve binding of Nb 3E11.

### 4.8. Hydrogen/deuterium exchange mass spectrometry

Hydrogen/deuterium exchange mass spectrometry (HDX-MS) experiments were carried out using an automated HDX robot (LEAP Technologies) coupled to an M-Class Acquity LC and HDX manager (Waters Ltd) [74]. All samples were diluted to 10 µM in equilibration buffer (10 mM potassium phosphate buffer pH 7.0) prior to analysis. 30 µl sample was added to 135 µl deuterated buffer (10 mM potassium phosphate buffer pH 7.0) and incubated at 4°C for 0.5, 2, 5 or 10 min. Following the labelling reaction, samples were quenched by adding 50 µl of the labelled solution to 100 µl quench buffer (50 mM potassium phosphate, 0.05% DDM pH 2.2) giving a final quench pH ∼ 2.5. 50 µl of quenched sample (*ca* 24 pmol) were passed through an immobilized ethylene bridged hybrid (BEH) pepsin column (Waters Ltd) at 500 µl min^−1^ (20°C) and a VanGuard Pre-column Acquity UPLC BEH C18 (1.7 µm, 2.1 mm × 5 mm, Waters Ltd) for 3 min in 0.3% formic acid in water. The resulting peptic peptides were transferred to a C18 column (75 µm × 150 mm, Waters Ltd) and separated by gradient elution of 0–40% MeCN (0.1% v/v formic acid) in H_2_O (0.3% v/v formic acid) over 7 min at 40 µl min^−1^. Trapping and gradient elution of peptides were performed at 0°C. The HDX system was interfaced to a Synapt G2Si mass spectrometer (Waters Ltd). HDMSE and dynamic range extension modes (data independent analysis (DIA) coupled with IMS separation) were used to separate peptides prior to CID fragmentation in the transfer cell. HDX data were analysed using PLGS (v3.0.2) and DynamX (v3.0.0) software supplied with the mass spectrometer. Restrictions for identified peptides in DynamX were as follows: minimum intensity, 1000; minimum products per MS/MS spectrum, 5; minimum products per amino acid, 0.3; maximum sequence length, 25; maximum error, 5 ppm; file threshold, 3/3.

### 4.9. Cell culture and transfection

MIA PaCa-2 (ATCC CRL-1420), B16-F1 (ATCC CRL-6323) and SK-OV-3 (ATCC HTB-77) cells were routinely cultured in Dulbecco's modified Eagle's medium (Gibco) supplemented with 10% fetal bovine serum (Gibco), 1% penicillin–streptomycin (100 U ml^−1^, Gibco), 1% l-glutamine (2 mM, Gibco) (complete medium) supplemented with 0.1 mg ml^−1^ Primocin (InvivoGen). Cells were maintained at 37°C in a 5% CO_2_ atmosphere and tested negative for mycoplasma contamination.

Adherent cells were transfected with Lipofectamine 2000 DNA transfection reagent (Invitrogen) according to the manufacturer's instructions. For each 9.5 cm^2^ well of adherent cells, 1 µg of DNA was mixed with 5 µl of Lipofectamine 2000 in Opti-MEM reduced serum medium (ThermoFisher) and incubated for 20 min at RT. Cells were washed with Opti-MEM medium and incubated with the DNA–Lipofectamine mixture for 4 h at 37°C with 5% CO_2_. Thereafter, the DNA–Lipofectamine mixture was removed and replaced with complete medium and incubated overnight at 37°C with 5% CO_2_.

### 4.10. Confocal microscopy

Super-resolution confocal microscopy was performed using a Zeiss 880 LSM equipped with Airyscan, a Plan-Apochromat 63x/NA 1.4 Oil DIC M27 objective and 488 nm and 561 nm laser lines in frame mode with 1.8× or 5× zoom. Live cells were maintained in an environmental chamber at 37°C and 5% CO_2_. Images were acquired using Airyscan and Airyscan Fast modes in Zen Black Software (v2.3 SP1) and Airyscan processed in Zen Blue Software (v.2.3 Desk). Cells were categorized discretely based on the number of filopodia (0–5, 6–10 or greater than 10). Fluorescence intensity quantification was performed in Fiji (ImageJ) using the Plot Profile plugin.

For nanobody dynamics experiments, cells transfected with nanobody-GFP and mCherry-Fascin-1 were imaged as above in Airyscan Fast mode. To minimize phototoxicity, cells were imaged every 5 s for 2.5 min, and filopodia lifetime quantified using the Manual Tracking plugin in Fiji (ImageJ).

### 4.11. Widefield microscopy

To assess cell proliferation, cells were imaged with an Incucyte Zoom or S3 live-cell imaging system (Sartorius) every 1 h for up to 3 days. Cell confluence was quantified using Incucyte Zoom software (v. 2018A) or Incucyte S3 software (v. 2022A). The experiment was repeated at three times to generate biological replicates.

### 4.12. Statistical analysis

Graphs were plotted and statistical analysis was performed with Prism 7 (GraphPad). The null hypothesis in each case was that there was no difference between the control conditions and Nb 3E11 treatment. To determine the appropriate statistical test to perform, the data were checked for a normal distribution using Shapiro–Wilk and Kolmogorov–Smirnov tests. If the data distributions did not satisfy the normality tests, non-parametric statistical tests were used to test the null hypothesis.

## Data Availability

The coordinates of the Fascin-1/Nb 3E11 complex structure have been deposited in the RCSB PDB database (www.rcsb.org) with the identifier 7ZAU. The mass spectrometry proteomics data have been deposited to the ProteomeXchange Consortium via the PRIDE [75] partner repository with the dataset identifier PXD038713. All other data needed to evaluate the conclusions in the paper are present in the paper or the electronic supplementary material. Supplementary material is available online [76].
